# Immunogenic Salivary Proteins of *Triatoma infestans*: Development of a Recombinant Antigen for the Detection of Low-Level Infestation of Triatomines

**DOI:** 10.1371/journal.pntd.0000532

**Published:** 2009-10-20

**Authors:** Alexandra Schwarz, Stefan Helling, Nicolas Collin, Clarissa R. Teixeira, Nora Medrano-Mercado, Jen C. C. Hume, Teresa C. Assumpção, Katrin Marcus, Christian Stephan, Helmut E. Meyer, José M. C. Ribeiro, Peter F. Billingsley, Jesus G. Valenzuela, Jeremy M. Sternberg, Günter A. Schaub

**Affiliations:** 1 School of Biological Sciences, Zoology Building, University of Aberdeen, Aberdeen, United Kingdom; 2 Group Zoology/Parasitology, Ruhr-Universität Bochum, Bochum, Germany; 3 Medizinisches Proteom-Center, Ruhr-Universität Bochum, Bochum, Germany; 4 Vector Molecular Biology Unit, Laboratory of Malaria and Vector Research, National Institute of Allergy and Infectious Diseases, National Institutes of Health, Rockville, Maryland, United States of America; 5 Laboratory of Chagas Disease and Immunoparasitology, Biology Department–Facultad de Ciencias y Tecnología, Universidad Mayor de San Simón, Cochabamba, Bolivia; 6 International Studies of Malaria and Entomology Section, Laboratory of Malaria and Vector Research, National Institute of Allergy and Infectious Diseases, National Institutes of Health, Rockville, Maryland, United States of America; 7 Laboratory of Host-Parasite Interface, University of Brasilia, Brasilia-DF, Brazil; 8 Sanaria, Rockville, Maryland, United States of America; National Yang-Ming University, Taiwan

## Abstract

**Background:**

Triatomines are vectors of *Trypanosoma cruzi*, the etiological agent of Chagas disease in Latin America. The most effective vector, *Triatoma infestans*, has been controlled successfully in much of Latin America using insecticide spraying. Though rarely undertaken, surveillance programs are necessary in order to identify new infestations and estimate the intensity of triatomine bug infestations in domestic and peridomestic habitats. Since hosts exposed to triatomines develop immune responses to salivary antigens, these responses can be evaluated for their usefulness as epidemiological markers to detect infestations of *T. infestans*.

**Methodology/Principal Findings:**

*T. infestans* salivary proteins were separated by 2D-gel electrophoresis and tested for their immunogenicity by Western blotting using sera from chickens and guinea pigs experimentally exposed to *T. infestans*. From five highly immunogenic protein spots, eight salivary proteins were identified by nano liquid chromatography-electrospray ionization-tandem mass spectrometry (nanoLC-ESI-MS/MS) and comparison to the protein sequences of the National Center for Biotechnology Information (NCBI) database and expressed sequence tags of a unidirectionally cloned salivary gland cDNA library from *T. infestans* combined with the NCBI yeast protein sub-database. The 14.6 kDa salivary protein [gi|149689094] was produced as recombinant protein (r*Ti*SP14.6) in a mammalian cell expression system and recognized by all animal sera. The specificity of r*Ti*SP14.6 was confirmed by the lack of reactivity to anti-mosquito and anti-sand fly saliva antibodies. However, r*Ti*SP14.6 was recognized by sera from chickens exposed to four other triatomine species, *Triatoma brasiliensis*, *T. sordida*, *Rhodnius prolixus*, and *Panstrongylus megistus* and by sera of chickens from an endemic area of *T. infestans* and Chagas disease in Bolivia.

**Conclusions/Significance:**

The recombinant r*Ti*SP14.6 is a suitable and promising epidemiological marker for detecting the presence of small numbers of different species of triatomines and could be developed for use as a new tool in surveillance programs, especially to corroborate vector elimination in Chagas disease vector control campaigns.

## Author Summary

Chagas disease, caused by *Trypanosoma cruzi*, is a neglected disease with 20 million people at risk in Latin America. The main control strategies are based on insecticide spraying to eliminate the domestic vectors, the most effective of which is *Triatoma infestans*. This approach has been very successful in some areas. However, there is a constant risk of recrudescence in once-endemic regions resulting from the re-establishment of *T. infestans* and the invasion of other triatomine species. To detect low-level infestations of triatomines after insecticide spraying, we have developed a new epidemiological tool based on host responses against salivary antigens of *T. infestans*. We identified and synthesized a highly immunogenic salivary protein. This protein was used successfully to detect differences in the infestation level of *T. infestans* of households in Bolivia and the exposure to other triatomine species. The development of such an exposure marker to detect low-level infestation may also be a useful tool for other disease vectors.

## Introduction

Control programs for Chagas disease in South America, such as the ‘Southern Cone Initiative’ have relied mainly upon vector control using insecticide spraying [1]. These campaigns have reduced the distribution of *T. infestans* to an area of 14.6% of the initial endemic area. However, especially the Gran Chaco region (Bolivia, Argentina, Paraguay), Andean Bolivia, western Argentina and a small area in south Peru are now still harbouring significant vector populations, some of these regions with sylvatic foci of *T. infestans* [1]–[3]. In controlled and Chagas disease free areas, i. e. free of vector-borne transmission, such as Argentina and Uruguay, surveillance activities have been greatly reduced allowing the re-establishment of *T. infestans* [4]. Moreover, after elimination of domestic vectors, peridomestic or sylvatic bug populations and species persist and may replace the former domestic populations due to changes in the ecological balance [5].

Current methods to assess the prevalence and intensity of triatomine bug infestations in domestic and peridomestic sites involve timed manual collections using an irritant spray or artificial shelter units. These methods are costly, require skilled staff, and usually lack the sensitivity and precision necessary for detection of low-density populations. Additionally, current methods are too expensive for large-scale surveillance campaigns and are not easily adaptable to many peridomestic sites [6]–[8]. Thus, new methodologies are required to detect re-emerging *T. infestans* populations and for long-term monitoring of previously endemic regions for Chagas disease [9].

Hematophagous arthropods have evolved a wide range of salivary anti-hemostatic compounds such as anti-coagulants, anti-histamines, vasodilators and inhibitors of platelet aggregation, sodium channel blockers, immunosuppressors, pore forming molecules and complement inhibitors that are injected into the host when feeding on blood to overcome host defence mechanisms (hemostasis, inflammation, immunity) [10]–[15]. Salivary proteins can elicit humoral immune-responses in their hosts [16]–[20]. The detection of antibodies to salivary antigens has been used as an epidemiological tool and biological marker of exposure to disease vectors including mosquitoes, ticks, sand flies and tsetse flies [19], [21]–[27]. The humoral immune response to salivary proteins of triatomines were studied in chickens, guinea pigs, mice, rabbits and humans; the latter studies using saliva and focusing on epidemiology [16], [28]–[31].

Recently, we described the anti-saliva immune responses of chickens and guinea pigs which had been experimentally exposed to *T. infestans* [32]. Antibody responses were detected as soon as two days after the first bug bites. Salivary antigens of 14 and 21 kDa were recognized by all chicken sera and a 79 kDa protein by all guinea pig sera. Sera from animals naturally exposed to triatomines in Bolivia also reacted with these antigens. In the present paper we describe the development of a highly sensitive exposure assay resulting in a specific recombinant salivary antigen to be used as an epidemiological tool for Chagas disease surveillance.

## Materials and Methods

### Insects: Origin, maintenance, saliva and salivary gland protein collection

*Triatoma infestans*, *T. brasiliensis*, *T. sordida*, *Rhodnius prolixus and Panstrongylus megistus* were reared at 27±1°C, 60–70% relative humidity, with a 16/8-h light/dark cycle and were fed on chickens [33]. *T. infestans* originated from a domestic population from Northern Chile, the Cachiyuyo village (29°1′48.90″S, 70°53′55.53″W, 808 m), at the border of the provinces Atacama and Coquimbo [34],[35]. *T. brasiliensis* and *T. sordida* were originally collected from a chicken house from Sítio do Cleniro and Bairro Sosó, state of Piauí, Brazil (GAS). *R. prolixus* originated from San Juan de Arama, Meta, Colombia (obtained from A. D'Alessandro-Bacigalupo) and *P. megistus* from Minas Gerais, Brazil (obtained from J. Jurberg, Departamento de Entomologia, Instituto Oswaldo Cruz, Rio de Janeiro, Brazil). All experiments were performed with pooled saliva obtained from about 300 fifth instars and adults using capillary pipettes [36]. Typically 0.5–1 µl saliva were obtained from each bug. The saliva was desalted with a 4 kDa cut-off centrifugal concentrator Fugisep-Mini (Intersep), and the protein concentration was determined using a BCA Protein Assay Kit (Perbio Science) according to the manufacturer's instructions. Aliquots of saliva, containing 30 µg protein/µl, were stored at −80°C.

Adult *Anopheles freeborni* from Marysville (California, USA), *Aedes aegypti* (Liverpool black eye strain, UK) and *Culex quinquefasciatus* from Vero Beach (Florida, USA) were maintained at 28°C, 75% relative humidity and a 12/12 h light/dark cycle. They were provided with 10% sucrose solution for maintenance and blood-fed on anesthetized BALB/c mice [37],[38]. *Lutzomyia longipalpis* (Jacobina strain, Brazil) were reared according to Modi and Tesh [39] with modifications. Briefly, adult sand flies were maintained at 26°C and 70% relative humidity with a 14/10 h light/dark cycle and fed either with 30% fructose solution or on anesthetized C57Bl/6 mice [32].

### Immune sera

The early IgG-response and serial challenges of chickens and guinea pigs by *T. infestans* have been described previously [32]. Briefly, for the early response five chickens were exposed to starved adult *T. infestans* (5 per chicken) for 1 h and blood samples were taken daily for five days. For serial challenge, 12 chickens and 10 guinea pigs were exposed every two weeks over a period of 1 h or 30 min and for 19 or 23 weeks, respectively, to a low (5 adults) or a high (5 adults and 20 fourth and fifth instars) number of *T. infestans*. Sera from animals taken prior to the first feeding served as negative control. For the positive control, sera were pooled from chickens which had been used for routine maintenance of triatomines for at least six months.

Groups of three chickens were exposed weekly either to five adults (*T. brasiliensis*, *T. sordida*, *R. prolixus* or *P. megistus*) or *An. freeborni*, *Ae. aegypti* or *Cx. quinquefasciatus* (approx. 500 insects/animal/expsoure) for one month. Triatomines were allowed to feed 1 h and mosquitoes about 30 min (until about 90–100% had fed). Blood samples were collected before and always five days after exposure (triatomines) or at the end (mosquitoes), and the exposure sera were pooled [32]. Pooled serum was also prepared after one month from five mice exposed weekly for 30–45 min to *L. longipalpis* (approx. 100 insects/animal/exposure).

From September to November 2007, *T. infestans* were collected at peridomestic sampling sites and blood samples from animals were only taken if either 1–12 bugs (low exposure group) or ≥100 bugs (high exposure group) were collected by 3–5 persons within 30–60 min. Blood samples from 28 chickens (taken from the brachial vein) and 26 guinea pigs (taken from the ear vein) were collected from 16 out of 17 households in the following rural villages in the Department of Cochabamba: Sipe Sipe (17°27′2.78″S, 66°21′38.91″W, 2555 m; 2 out of 3 households colonized), Lipez (17°33′47.12″S, 66°15′27.643″W, 2542 m; 5 households), Chajra Corral (18°1′18.30″S, 64°55′25.157″W, 1796 m; 3 households), Pampas (18°3′26.812″S, 64°54′35.01″W, 1708 m; 3 households), Peña Colorada (18°10′5.288″S, 64°52′0.309″W, 1583 m; 1 household) and Arpita (17°33′51.62″S, 66°4′15.049″W, 718 m; 2 households). The blood was centrifuged at 10,000×g for 10 min at room temperature, and the sera were stored at −20°C.

### Ethics statement

All animal procedures described in this study and carried out at the Ruhr-Universität Bochum were approved by the Landesamt für Natur, Umwelt und Verbraucherschutz Nordrhein-Westfalen, Recklinghausen, Germany. All animal studies carried out at the The National Institute of Allergy and Infectious Diseases (NIAID) were approved by the Animal Care and Use Committee at NIAID, Bethesda, MD, USA. In Bolivia the blood was taken by veterinarians of the Faculty of Veterinary, Universidad Mayor de San Simón, Cochabamba, Bolivia.

### 2D-gel electrophoresis and Western blots

Salivary gland proteins of *T. infestans* were separated by isoelectric focusing (IEF) using a Multiphor II Electrophoresis Unit (GE Healthcare), pH 4–10 [40]. Saliva (40 µg protein) was dissolved in reducing buffer containing 1% ß-mercaptoethanol and 0.5% Ampholine carrier ampholytes, pH 3.5–10 (GE Healthcare). Immobilized pH gradient (IPG) strips (7 cm, pH 4–10) were rehydrated with the protein solution and the proteins focused at 10°C using the following gradient: 0–200 V, 2 mA, 5 W for 5 min, then 200–3500 V, 1 mA, 3 W for 90 min and 3500 V constantly, 1 mA, 3 W for 60 min. For the second dimension electrophoresis, the IPG strips were equilibrated in Laemmli loading buffer [41]. The strips were placed onto 15% SDS-PAGE gels and the proteins separated according to their molecular weight using a Hoefer SE 600 apparatus (GE Healthcare). Ten independent samples of saliva were separated to recognize differences in the protein profile.

Gels were stained either with colloidal Coomassie blue or silver nitrate with modifications to ensure mass spectrometry-compatibility [42],[43]. Molecular weights were calculated with reference to the mobility of standard proteins (Prestained Protein Marker, New England Biolabs) included in one lane of the gel using the software ImageMaster 2D Elite, version 4.3 (GE Healthcare).

Western blot analyses were carried out with replica gels of the 2D-gel electrophoresis using individual sera from chickens and guinea pigs exposed to low and high numbers of triatomines in the long-term exposure study [32]. The Western blot images were compared to silver stained 2D-gels in order to determine the recognized salivary gland proteins.

### Protein identification by mass spectrometry (MS)

Salivary proteins of *T. infestans* which were recognized by immune animal sera in the 2D-Western blot experiments were identified by mass spectrometry. Colloidal Coomassie stained protein spots were decolorised using alternating solution A (20 mM ammonium bicarbonate) and solution B (10 mM ammonium bicarbonate, 50% acetonitrile). Silver stained spots were decolorised with 50 mM sodium thiosulfate and 15 mM potassium hexacyanoferrate (III) followed by washing with solution A and B [44]. Proteins from all spots were trypsin-digested and extracted [44].

The peptides were subject to liquid chromatography-electrospray ionization-tandem mass spectrometry (nanoLC-ESI-MS/MS) analysis with collision induced dissociation experiments using the Ultimate 3000 HPLC system (Dionex LC Packings) coupled online to the HCTultra PTM Discovery System ion trap mass spectrometer (Bruker Daltonics) [45]. For protein identification, the MS output files (mgf format) were stored in the Proteinscape database (Bruker Daltonics) and the MS/MS data were compared to the protein sequences of the NCBI database (Update: 07/05/2007) using both the Mascot and Sequest algorithms. To improve the protein identification the MS/MS data were also compared to expressed sequence tags of a unidirectionally cloned salivary gland cDNA library from *T. infestans* combined for statistical reasons to the yeast protein sub-database of the NCBI database (TaxID 4932, 07/05/2007) [46]–[48]. The following search parameters were selected: peptide mass accuracy of 0.6 Da (mono-isotopic), fragment mass accuracy of 0.2 Da (mono-isotopic), variable modification due to oxidation of methionine, acrylamide modification of cysteine, two maximal missed cleavage sites in case of incomplete protease digests. As database searches did not consider all possible types of fragment ions, the quality of the fragment ion spectra explaining identified peptides was analysed using the ESI Compass 1.3, DataAnalysis 4.0 software (Bruker Daltonics) and by theoretical fragmentation with the MS-Product software tool (http://prospector.ucsf.edu/cgi-bin/msform.cgi?form=msproduct). For positive protein identification, a minimum of two unique peptides, adequately explained by additional manual interpretation of the respective fragment ion spectra, and at least 5% sequence coverage of the respective protein were required.

### Heterologous synthesis and HPLC purification of *T. infestans* salivary proteins

Complementary DNAs from the *T. infestans* library encoding the salivary antigens were amplified by PCR, cloned into the VR2001-TOPO plasmid and purified as described previously (for primer sequences see Table S1) [47],[49]. To aid purification, a six histidine-tag coding sequence was added to the C-terminus of the cDNAs by PCR in a single step using reverse primers ending with the histidine-tag and the stop codon. Plasmids were analyzed for their correct insert and the insert orientation by sequencing using a CEQ 2000 DNA sequencer (Beckman Coulter) as previously described [49].

Plasmid purification was carried out using the GenElute HP Endotoxin-Free Plasmid Megaprep Kit (Sigma-Aldrich) according to manufacturer's instructions. DNA was concentrated with a Centricon plus-20 centrifugal filter device (Millipore) of a 100 kDa cut-off. After measurement of the concentration using the NanoDrop spectrophotometer, the DNA solution was sterile filtered using a 0.22 µm Millex-GS Filter Unit (Millipore) and stored at −80°C.

FreeStyle 293-F Cells (Invitrogen) (1×10^6^ cells/ml) were transfected with plasmids coding for salivary gland proteins following the manufacturer's instructions and incubated for 72 h at 37°C, 8% CO_2_ on a stirrer plate with the propeller of the flask rotating at 135 rpm. Forty-eight hours after transfection, cells were centrifuged and the supernatant filtered through a 0.8 µm filter unit (Nalgene Labware). The supernatant was concentrated using an Amicon ultrafiltration device with a 10 kDa cut-off membrane (Millipore) in presence of Buffer A (20 mM NaH_2_PO_4_, 20 mM Na_2_HPO_4_, 500 mM NaCl, pH 7.4). After concentration, the expressed recombinant proteins were dialysed using Slide-A-Lyzer Dialysis Cassettes (3 kDA cut-off, Pierce) against PBS at 4°C overnight.

The proteins were purified by HPLC using a HiTrap Chelating HP column (GE Healthcare). The concentrated supernatant was loaded onto a prepared HiTrap Chelating HP column (GE Healthcare) and the column connected to a Summit HPLC System with a P680 HPLC pump and a PDA-100 photodiode array detector (Dionex). The column was washed for at least 30 min with buffer A and the following gradient was used to elute the proteins at a flow rate of 1 ml/min: 0–10 min buffer A, from 10–25 min buffer B (buffer A plus 50 mM imidazole, pH 7.4,), 25–45 min 80% buffer B and 20% buffer C (buffer A plus 500 mM imidazole, pH 7.4), 45–105 min buffer C and 105–106 min buffer A. The elution of the proteins was detected at 280 nm, and protein fractions were collected every minute in a 96 deep well microtiter plate using a Foxy 200 fraction collector (ISCO).

The purity of the fractions containing the recombinant proteins was tested by SDS-PAGE using a 4–12% NuPAGE Novex 4–12% Bis-Tris gel and the XCell *SureLock* Mini-Cell electrophoresis system (Invitrogen). The gel was silver stained using the SilverQuest silver staining kit (Invitrogen). Only fractions containing the recombinant proteins were pooled and dialysed against PBS, and the purity of the proteins was tested again by SDS-PAGE and silver staining. The protein concentration was measured using the NanoDrop spectrophotometer. Only the 14.6 kDa recombinant salivary protein was obtained in sufficient amount and stored at −80°C.

### Deglycosylation of the recombinant salivary protein of *T. infestans*

The 14.6 kDa recombinant salivary protein of *T. infestans* was deglycosylated using the enzymatic deglycosylation kit Glyko according to the manufacturer's instructions (ProZyme Inc.). Three aliquots each containing 5 µg recombinant protein were incubated with N-Glycanase PNGase F, a mixture of PNGase F and Sialidase A or a mixture of PNGase F, Sialidase A and O-Glycanase, respectively. After incubation for 3 h at 37°C, the effect of deglycosylation was assessed by SDS-PAGE, and the peptides visualised by SimplyBlue SafeStain solution (Invitrogen).

### ELISA

Concentrations of anti-saliva IgG in pooled chicken and guinea pig sera from the long-term *T. infestans* exposure study were measured by ELISA using 0.5 µg recombinant salivary protein per well of a 96-well microtitre plate (Immunolon, Nunc, Wiesbaden, Germany) as previously described [32]. Individual serum samples from Bolivia were tested either using 0.5 µg recombinant salivary protein or crude saliva of *T. infestans* per well as the antigen. Responses to challenge with other hematophagous insects to measure cross-reactivity used capture ELISA with recombinant salivary gland protein of *T. infestans* as described above.

### Data analysis

Analysis of data was performed using SigmaStat 3.1 (Systat Software Inc.). Comparison of responses to bug exposure in chickens and guinea pigs in the long-term study was carried out using a Friedman Repeated Measures Analysis of Variance on Ranks (One Way RM ANOVA) with a Pairwise Multiple Comparison Procedure (Tukey test) as these data were normally distributed. In the other experiments, depending on the distribution of the ELISA test data, either an Unpaired t-test or a Mann-Whitney Rank Sum test was carried out to compare ELISA assay results using the recombinant salivary protein or the crude saliva of *T. infestans* analysed with sera from former laboratory studies or field collections sites in Bolivia [32]. The level of significance for all tests was *p*≤0.05.

Initially, the amino acid sequence of the protein was compared to the NCBI protein database to identify species with putative similar proteins. Found sequences were aligned using Clustal X 2.0.3, and graphically displayed using BioEdit 7.0.9.0 [50],[51]. The presence of a signal secretion peptide was predicted by SignalP 3.0 [52].

## Results

### Salivary antigens of *T. infestans* recognized by host antibodies

**Figure 1:**
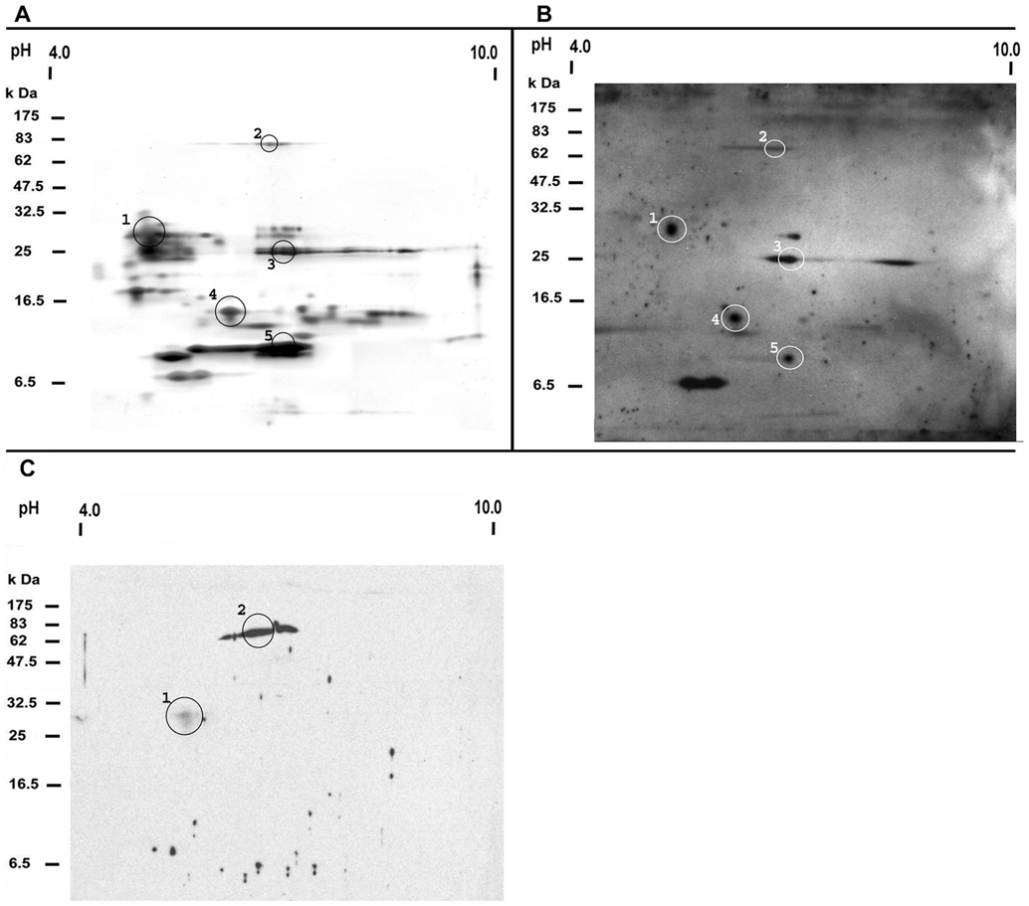
Immunogenic proteins in *T. infestans* saliva. Salivary proteins were resolved by 2D-gel electrophoresis (A) and 2D-Western blot (B, C) of the saliva of *T. infestans*. The molecular weight standard and the pI range (from pH 4 to 10) are included in each panel. Protein spots which were recognized by all chicken (B) and all guinea pig sera (C) exposed to *T. infestans* are encircled and numbered (1–5 or 1–2) on the gel and blot. Corresponding protein spots of the 2D-gel and the 2D-Western blot are labelled with the same number.

2D-gel electrophoreses of *T. infestans* salivary proteins revealed consistently (n = 10) that most proteins were present between pH 4.6 and 7.3 and between 25 and 31 kDa (Fig. 1A). In 2D-Western blots using serum from *T. infestans* challenged chickens and guinea pigs, four different proteins (protein spots 1, 3, 4 and 5) of 30, 26, 14 and 12 kDa reacted with sera of all *T. infestans* challenged chickens (Fig. 1B), while a 79 kDa protein (spot 2) was recognized by 8 out of 10 challenged animals. Sera from all challenged guinea pigs reacted with a 79 kDa protein (spot 2) and weakly recognized a 30 kDa protein (spot 1) (Fig. 1C).

**Figure 2:**
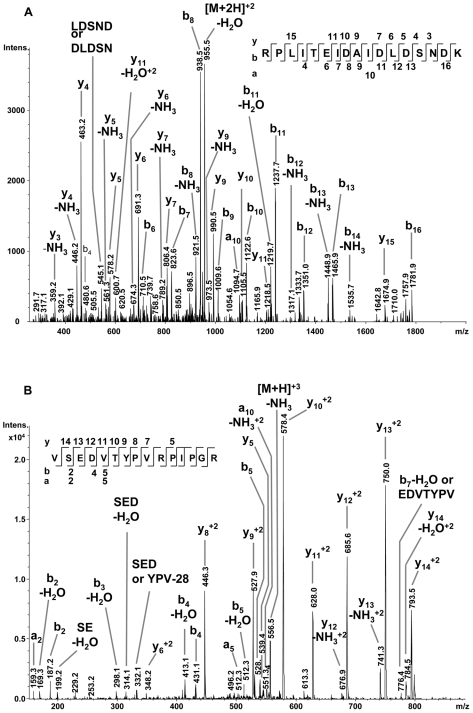
NanoLC-ESI-MS/MS analysis of the 14.6 kDa salivary secreted protein of *T. infestans* (gi|149689094). MS/MS spectra are shown for the two unique peptides of the identified protein: (A) RPLITEIDAIDLDSNDK with the doubly charged precursor ion mass/charge ratio (m/z) 964.5 Da and (B) VSEDVTYPVRPIPGR with the doubly charged precursor ion m/z 562.6 Da. The y-(C-terminus of the peptide), b-(N-terminus of the peptide) and a-(N-terminus of the peptide) fragment ions are highlighted in the spectra and at the peptide sequences.

Partial peptide sequences from these five protein spots were identified by mass spectrometry and compared to the NCBI database and to a salivary gland cDNA library from *T. infestans*. Eight different protein matches were identified within these spots (Table 1). As an example, Fig. 2A and 2B present MS/MS spectra of two unique peptides of the 14.6 kDa salivary secreted protein [gi|149689094] from spot 4. In all cases there was a difference between theoretical and measured pI, and also the theoretical molecular weights derived from the cDNA sequence differed from the molecular weight determined by the 2D-gel; protein spot 4 contained not only a protein of 14.6 kDa but also a 21.4 kDa salivary protein. This also applies to all other protein spots except for protein spot 1 (Table 1).

**Table 1:** Protein identification of salivary proteins of *T. infestans* by mass spectrometry.

Spot no.	Proteinno.	Data-base#	Accession number	Protein name	Protein family	MW* (kDa)	pI* (kDa)	MS/MS analysis		
								Mascot/Sequest score	Protein sequence coverage (%)	Peptide sequence
1	1	NCBI	gi|149898926	truncated pallidipin-like	triabin	29.5/30	4.2/5.2	77.2/7.5	25.8	EQVEDGEEGALIEK
				lipocalin precursor				77.7/6.9		FFEGDWSLTYSTR
								49.7/8.1		HYGLTMDEFITR
								40.2/9.5		KEQVEDGEEGALIEK
								42.9/8		TDEEYVK
								37.8/7		VTESTINR
								31.8/8.4		YGSMVGFDPK
	2	dbEST	gi|148469123	truncated pallidipin-like	triabin	29.5/30	4.3/5.2	96.3/9.3	36.7	EQVEEGEEGALIEK
				lipocalin precursor				57.5/6.8		FFEGDWSLTYSTK
								56.7/6.2		GGNGAQEEKGETGAQEEK
								49.7/8.1		HYGLTMDEFITR
								73.9/7.6		LIEYGQNQCQR
								42.9/8		TDEEYVK
								37.8/6.2		VTESTINR
								57.6/10.8		YGSMTGFEPNR
2	3	NCBI	gi|34481604	79 kDa salivary apyrase	5′nucleotidase	62.5/79	9.5/6.4	52.2/9.7	14.0	GANLIFAVGHSGINIDK
				precursor				43.9/13.1		GVNIAVIGYMTPDTK
								37.6/5.9		LTLLHTNDMHSR
								N.F./4.3		VFIPNVLK
								62.8/13.6		WTDVPITLINGGSIR
	4	dbEST	gi|148468017	truncated 79 kDa	5′nucleotidase	18.8/79	9.5/6.4	44/5.4	43.4	EQVYEEVDLK
				salivary apyrase				83/8.5		GKGSGEFLQFSGLK
				precursor				42.8/5.9		GSGEFLQFSGLK
								28.4/7.5		IIISKPLPTLNLNNPK
								30.9/4.5		LSMPGSTLK
								59.5/11.8		YLEQMSPVYTGLQAR
								24.8/N.F.		YYTIAEDK
3	5	dbEST	gi|148468913	truncated unknown		18.2/26	6.3/6.6	33.1/4.8	84.7	AGIEIQK
				salivary protein				31.6/7.9		AGKDLYK
								N.F./4.4		DIEKGWNK
								27.6/5.2		DVGEELEK
								N.F./5.8		EIEKGFEDVGSELEK
								36.3/5.8		EIIEKIENGWK
								N.F./7.9		FGKDIEK
								N.F./6.6		FGKDIEKGWNK
								24.1/5.6		FGKEIEK
								42.1/9.1		FGKEIEKGFEDVGSELEK
								N.F./11.4		FKFNNPIPK
								30.9/8.1		FNNPIPK
								67.7/7.8		FSHHHEHAPESEGIITISIE
								32.6/10		DGNLKGFEDVGSELEK
								53.3/7		GFEDVGSELEKYGK
								38.1/6.2		GFEDVGSELEKYGKEVER
								46.1/9.2		GFKDVGEELEK
								N.F./8		GFKDVGEELEKVGK
								N.F./10.8		IEEGFRDFGQK
								N.F./5		IENGWK
								N.F./4.5		IENGWKQFEKDTK
								N.F./5.1		KIEEGFR
								26.1/9.7		KIEEGFRDFGQK
								44.4/5.8		YGKEVER
4	6	NCBI	gi|149898816	salivary lipocalin	triabin	21.4/14	7.6/6.1	26.9/N.F.	37.2	CTSFQDR
								29.5/10.4		DATNAYDAVCR
								31.1/N.F.		DYALAYR
								56.6/N.F.		DYALAYRCTSFQDR
								35.1/15.3		KPYTTTCSSVISPIPYGTIR
								38.9/4.6		SVHTGNLVLLQR
								53/5.2		TETADGSKATAGLK
	7	NCBI	gi|149689094	salivary secreted protein		14.6/14	10.3/6.1	N.F./7.8	24.8	RPLITEIDAIDLDSNDK
								43.4/8.3		VSEDVTYPVRPIPGR
5	8	NCBI	gi|149689054	salivary lipocalin	triabin	20.0/10	9.2/6.6	N.F./4.9	44.1	AKTDFSAK
								66/13.2		DDNYLVLSR
								57.5/7.7		FFTGTWFVSHVQK
								93.3/11.3		GYKDDNYLVLSR
								N.F./8.7		RFFTGTWFVSHVQK
								46.2/9.8		TSGGQVIPASLK
								62.6/7.3		TSSTVCQTFTASTPSEGK
								64.7/8.6		YIVEYTYQSHTGEQR

# After nanoLC-ESI-MS/MS analysis the MS/MS data were compared to the protein sequences of the NCBI database (Update: 07/05/2007) using both the Mascot and Sequest algorithms and to expressed sequence tags (ESTs) of a unidirectionally cloned salivary gland cDNA library from *T. infestans* combined for statistical reasons to the yeast protein sub-database of the NCBI database (TaxID 4932, 07/05/2007). All assembled ESTs of the salivary gland protein database are published in the NCBI dbEST. Calculations (e.g. molecular weight (MW), isoelectric point (pI) etc.) for proteins of the cDNA library correspond to the mature salivary protein. Thus, only nucleotide sequences from the start (including the signal peptide) to the stop codon were considered. Not found Mascot or Sequest scores are indicated (N.F.).

*: Theoretical MW/calculated MW and theoretical pI/calculated pI from 2D-PAGE analysis.

### Expression of recombinant salivary protein of *T. infestans*

**Figure 3:**
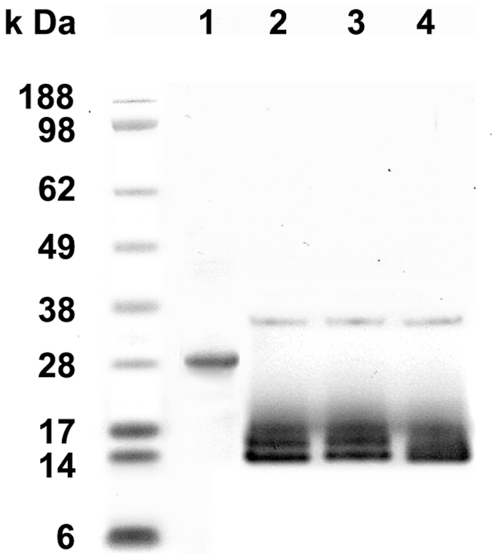
The salivary secreted protein of *T. infestans* (gi|149689094) after purification and deglycosylation. The His-tagged recombinant protein (r*Ti*SP14.6) was purified by HPLC (lane 1) and deglycosylated with PNGase F (lane 2), PNGase F and Sialidase A (lane 3) or PNGase F, Sialidase A and O-glycanase (lane 4). The molecular weight standard is given on the left in the graph.

Four out of eight identified salivary proteins of *T. infestans* were selected for recombinant expression. These were the truncated 79 kDa salivary apyrase precursor (spot 2), the truncated unknown salivary protein (spot 3), the salivary lipocalin and the salivary secreted protein (both in spot 4). Of these four proteins, the 14.6 kDa salivary secreted protein of *T. infestans* (spot 4) was successfully produced as recombinant protein in the mammalian expression system in sufficient amount and purified by HPLC (Fig. 3). All other proteins were expressed at very low levels and were not used for further analyses. SDS-PAGE analyses of the recombinant 14.6 kDa *T. infestans* salivary protein (r*Ti*SP14.6) (Fig 3, lane 1) gave an apparent molecular weight of 28 kDa. Enzymatic deglycosylation resulted in the expected molecular weight of 14 kDa on SDS-PAGE (Fig. 3, lanes 2–4).

### Antibody response in experimental challenge to rTiSP14.6

**Figure 4:**
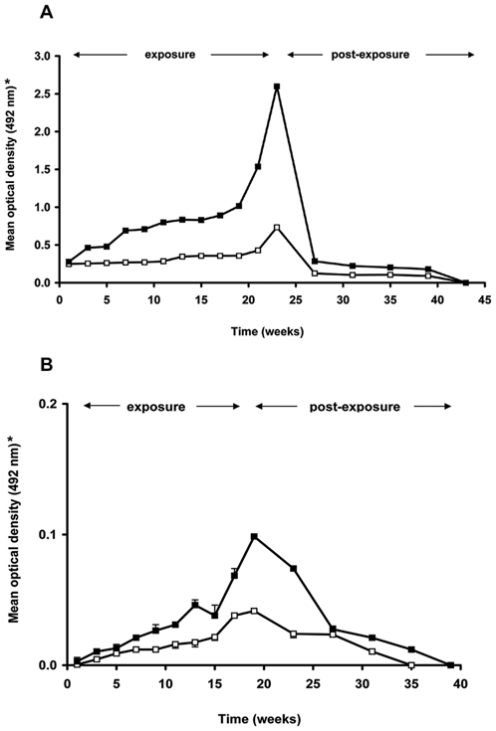
Evolution of serum IgG reactions to r*Ti*SP14.6 during exposure and post-exposure period. Reactivity to r*Ti*SP14.6 was measured in sera from chickens (A) and guinea pigs (B) using ELISA. (*) Data presented are mean and standard deviation of optical densities of two series of measurements (assayed in triplicate) using sera from the low (open squares) and high (closed squares) exposure groups (exposure to 5 or 25 *T. infestans* per feeding, respectively).

The earliest antibody reaction with r*Ti*SP14.6 was detectable from the second day onwards after a single challenge of five bugs per chicken (n = 5, mean O.D._492 nm_ = 0.032). In the long-term exposure study, chickens had detectable responses in the low (O.D._492 nm_ = 0.249) and high (O.D._492 nm_ = 0.279) exposure groups, at the first time point of sera collection (five days after the first exposure) (Fig. 4A). Subsequently, responses increased up to O.D._492 nm_ = 0.734 and O.D._492 nm_ = 2.598 for the low and high exposure groups, respectively, at the end of the 19 or 23 week exposure period. Overall, the antibody reaction measured with r*Ti*SP14.6 was significantly stronger in high exposure versus low exposure chicken sera (One Way RM ANOVA, *p*≤0.001). The serum reactivity declined rapidly during the first month post-exposure to O.D._492 nm_ = 0.125 (low exposure) and O.D._492 nm_ = 0.285 (high exposure). Afterwards, the serum reactivity remained stable for three months and then declined to zero. For all guinea pig sera, only a very weak reaction occurred in the low (maximum mean O.D._492 nm_ = 0.042) and high (maximum mean O.D._492 nm_ = 0.100) exposure groups, possessing no statistically significantly differences (*p*>0.05) (Fig. 4B). The intensity of the reaction also decreased rapidly after 20 weeks of exposure during four months post-exposure time.

**Figure 5:**
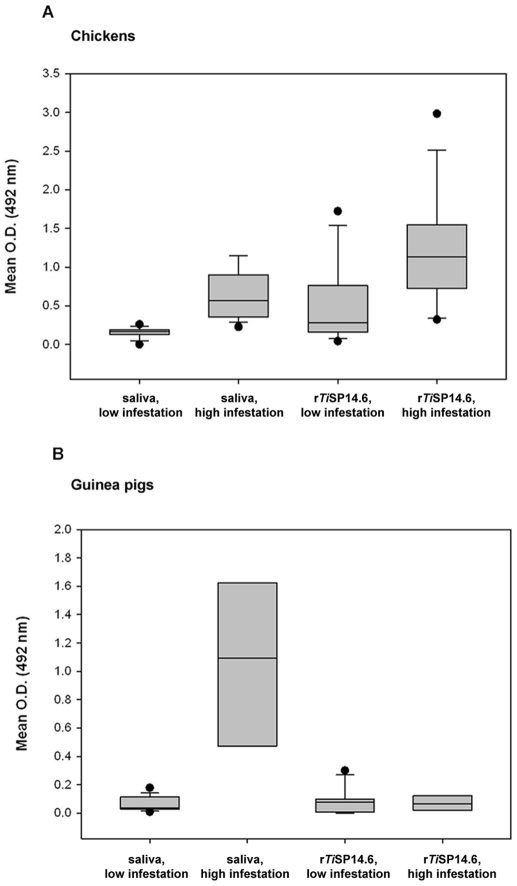
Reactions of *T. infestans* saliva and r*Ti*SP14.6 with chicken and guina pig sera from Bolivia. Chicken (A) and guinea pig (B) sera were collected in the Department of Cochabamba in households with a low (1–12 bugs) or high (≥100 bugs) infestation levels of triatomines from September to November 2007. Every ELISA assay was repeated and the final optical density determined by calculating the mean O.D._492 nm_ of the triplicate wells in two experiments, respectively, and subtracting the O.D._492 nm_ of the negative control. Boxes show median and 25th and 75th percentiles of mean ELISA optical densities for the different antigens and exposure groups. Whiskers show 10th and 90th percentiles. Dots represent outliers.

Antibody responses to crude saliva and r*Ti*SP14.6 of *T. infestans* were measured using sera from peridomestic hosts from villages with low and high *T. infestans* infestation densities (Fig. 5, for detailed antibody reactivities see Table S2). In the case of chickens, a significantly higher serum antibody response to both crude saliva (Mann-Whitney Rank Sum test, *p* = 0.001) and r*Ti*SP14.6 (Mann-Whitney Rank Sum test, *p* = 0.006) was detected in the high infestation households as compared to the low-infestation households (Fig. 5A). In the case of guinea pigs, this difference was only manifested in the serum antibody response to crude saliva (Mann-Whitney Rank Sum test, *p* = 0.001) (Fig. 5B).

### Specificity of rTiSP14.6 for estimating triatomine challenge

**Figure 6:**
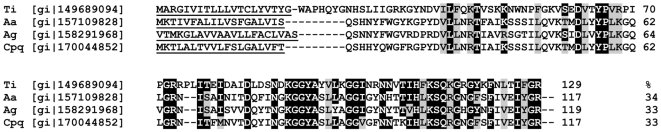
Alignment of predicted amino acid sequences of salivary proteins of *T. infestans* and mosquitoes. Similar peptide sequences of *T. infestans* (Ti, r*Ti*SP14.6), *Ae. aegypti* (Aa), A*n. gambiae* (Ag) and *Cx. pipiens quinquefasciatus* (Cpq) in the NCBI database were aligned using Clustal X 2.0.3. Accession numbers are shown in front of the sequence, percentages of sequence identities at the end. Signal peptides were predicted using Signal P 3.0 and are underlined at the beginning of the sequence. Identical residues with r*Ti*SP14.6 are marked by a black background and similar residues by a grey background [52].

The value of r*Ti*SP14.6 as a marker of triatomine challenge would be compromised if salivary antigens eliciting cross-reactive antibodies are expressed in other blood feeding insects feeding on the same host. A search of the NCBI database was therefore undertaken using the r*Ti*SP14.6 sequence and similar peptide sequences were found in *Ae. aegypti* (34% identity), *An. gambiae* (33% identity) and *Cx. pipiens quinquefasciatus* (33% identity) (Fig. 6). Challenge experiments with these hematophagous insects and other triatomine species were then undertaken to determine if cross-reactive antibodies were elicited. No antibody responses to r*Ti*SP14.6 were detected in sera from chickens exposed to *An. freeborni* (chickens, n = 3), *Ae. aegypti* (chickens, n = 3) and *Cx. quinquefasciatus* (chickens, n = 3) and in sera from mice (n = 5) exposed to *L. longipalpis*. Serum samples from chickens (n = 3) exposed only once to *T. brasiliensis* (mean O.D._492 nm_ = 0.075), *T. sordida* (mean O.D._492 nm_ = 0.078), *R. prolixus* (mean O.D._492 nm_ = 0.063) and *P. megistus* (mean O.D._492 nm_ = 0.093) all reacted with r*Ti*SP14.6. Comparing reactions of r*Ti*SP14.6 with sera from chickens exposed to other triatomines and the pooled serum of chickens to *T. infestans* the latter was 2.4 fold higher.

## Discussion

In this study, we used immune sera to identify and characterize immunogenic salivary proteins of *T. infestans* and made the first steps towards developing a novel immune-epidemiological tool to assess domestic and peridomestic infestations by *T. infestans*. In Western blots, sera of challenged chickens recognized salivary antigens of 30, 26, 14 and 12 kDa, and a 79 kDa antigen was recognized by sera from challenged guinea pigs. Differences between the molecular weights and pIs of these proteins estimated from the 2D-gels and the theoretical characteristics derived from the MS and cDNA sequences were most likely due to post-translational modifications, e.g. proteolytic cleavage, partial glycosylation, protein-protein complex formation [53].

In MS analyses, four proteins were identified as lipocalins. These comprise 55% of the putative secreted salivary proteins of *T. infestans* [47] and are also the most abundant secretory proteins in the saliva of *T. brasiliensis* and *R. prolixus* [54],[55]. Lipocalins are typically small extracellular proteins that possess a highly conserved eight-stranded antiparallel ß-barrel which forms a central hydrophobic ligand binding cavity. These secreted proteins are carriers of ligands involved in the retinol and pheromone transport, cryptic coloration, olfaction, prostaglandin synthesis and regulation of immune responses as well as the mediation of cell homoeostasis [56]–[58]. The salivary protein pallidipin, previously characterized in *T. pallidipennis*, is a lipocalin [59] and was highly immunoreactive at 30 kDa in the Western blots. This protein is an inhibitor of collagen-induced platelet aggregation and inhibits the release of ATP from platelets. Its effect on blood platelets is reversible, and it does not influence other types of platelet aggregation [59].

The 79 kDa salivary apyrase precursor from spot 2 is a member of the 5′-nucleotidase family and also a platelet aggregation inhibitor by hydrolysis of ATP or ADP to AMP [15], [60]–[63]. This glycosylated protein is frequently found in the saliva of different blood-feeding insects and ticks and was recently described in *T. infestans* [60],[64]. The apyrase was also detected in *T. brasiliensis*, but at 10-fold lower expression levels than in *T. infestans* [55],[65]. *R. prolixus* also possesses a similar apyrase [15],[66].

Four of eight identified proteins were selected for expression studies. The truncated 79 kDa salivary apyrase precursor [gi|148468017] of protein spot 2 was chosen because it was recognized by all guinea pig sera. The truncated unknown salivary protein [gi|148468913], the salivary lipocalin [gi|149898816] and the salivary secreted protein [gi|149689094] of spots 3 and 4 were chosen because they were recognized by chicken sera. Furthermore, the salivary lipocalin and the salivary secreted protein from protein spot 4 were available as full-length cDNA clones from the *T. infestans* cDNA library [47], increasing the likelihood that the expressed recombinant proteins would present the relevant epitopes. The salivary secreted protein was expressed successfully and named r*Ti*SP14.6. R*Ti*SP14.6 was insensitive to sialidase A and O-glycanase, but sensitive to peptide-N-glycosidase F. Therefore, the apparent 28 kDa MW was due to glycosylation with N-linked oligosaccharides. (In a subsequent MS analysis of r*Ti*SP14.6, two types of glycans were identified for one N-glycosylated site of r*Ti*SP14.6, biantennary and triantennary glycans linked to asparagine 105: NeuAcMan_5_GlcNac_4_, Man_8_GlcNAc_2_). Three N-glycosylated and no O-glycosylated sites were predicted for r*Ti*SP14.6 using the NetGlyc 1.0 and NetOGlyc 3.1 Server (detailed data not presented) [67],[68]. Such glycosylations are known for other *T. infestans* salivary proteins [16]. Differences in the glycosylation of r*Ti*SP14.6 from the native salivary secreted protein [gi|149689094] are probably due to it being expressed in a mammalian system rather than in its native insect salivary gland cell.

The suitability of r*Ti*SP14.6 as a tool for the immunological assay of exposure to *T. infestans* was demonstrated by several results. 1) The serum reactivity to r*Ti*SP14.6 increased with exposure time and gave a strong signal. 2) The sensitivity of r*Ti*SP14.6 was high enough to react with sera from chickens in low-infested households. 3) The weaker antibody reaction to r*Ti*SP14.6 in sera of guinea pigs than in chickens which also occurred in the field study, is no strong disadvantage, since chickens are found in all areas of the country, while guinea pigs only occur in limited areas restricted by the availability of alfalfa (*Medicago sativa*) which cannot be cultivated in the dry regions [69]. Since chickens are also present near or inside the houses in other countries of Latin America, r*Ti*SP14.6 is an excellent exposure marker for *T. infestans* (but see point 5 below). 4) Sera from hosts exposed to non-triatomine vectors (mosquito and sand fly species) failed to react with r*Ti*SP14.6, confirming the specificity of r*Ti*SP14.6 as an immune marker for triatomine challenge. Since in the previous investigation, cross reactions were also not found using bed bugs and ticks, false positive results in an epidemiological survey of triatomine distribution are less likely [32]. 5) A major advantage is the reaction of r*Ti*SP14.6 with sera of chickens challenged with *T. brasiliensis*, *T. sordida*, *R. prolixus* and *P. megistus*. Other triatomine species are capable of replacing *T. infestans*, and after control programs they may invade and colonize peridomestic sites; for example *T. brasiliensis* and *P. megistus* have replaced *T. infestans* in Brazil and *T. guasayana* may substitute for *T. infestans* in Argentina [1],[70],[71]. Although *Rhodnius* and *Triatoma*/*Panstrongylus* do not belong to the same tribe (Rhodniini: monophyletic or paraphyletic origin and Triatomini: polyphyletic or paraphyletic origin) within the polyphyletic Triatominae [72],[73], the native protein form of the cross reactive r*Ti*SP14.6 seems to be ubiquitous in the different triatomine families and probably is an evolutionary conserved protein (orthologous) in the Triatominae. These results are consistent with previous Western blot results showing all these species elicited antibodies to a 14 kDa salivary protein [32]. Therefore, r*Ti*SP14.6 is not *T. infestans* specific, but is suitable for detecting infestations by at least five species of triatomines. There seems to be a stronger reaction with sera challenged with *T. infestans* than with other triatomines which might allow a differentiation from these triatomine species using chicken sera. However, this needs to be verified in further investigations. Testing the more widespread use of r*Ti*SP14.6 as an exposure marker for triatomines other than *T. infestans* can best be evaluated by sera from chickens living in regions where other triatomines are found. Within such a survey, sera from other animals including dogs and cats, which are frequent domestic reservoir hosts of *T. cruzi*, can also be tested [4]. In a recent study, *T. infestans* showed a feeding preference for dogs over chickens and cats [74].

In conclusion, the aim of this study was to develop an epidemiological marker to detect low-level infestation of *T. infestans* in endemic countries after insecticide control activities. Out of eight different highly immunogenic *T. infestans* salivary proteins, a 14.6 kDa salivary protein of unknown function was successfully expressed recombinantly. It reacted strongly with sera from chickens exposed to a low number of *T. infestans* in both the laboratory and field. This recombinant protein will enable the immunological detection of low numbers of different triatomines, not only *T. infestans*, and hence could be developed as a sensitive surveillance tool to warn of exposure risk to triatomines and thereby for Chagas disease. The development of recombinant salivary proteins as epidemiological tools may also be useful for the control of other vectors such as mosquitoes, ticks, tsetse flies and sand flies and to understand the epidemiology of the diseases that they transmit [22], [75]–[78].

## Supporting Information

Alternative Language Abstract S1 Portuguese translation of the abstract by Dr. Eloi S. Garcia
(0.02 MB DOC)

Alternative Language Abstract S2 Spanish translation of the abstract by Dr. Ricardo E. Gürtler.
(0.02 MB DOC)

Table S1 Primer sequences used for PCR amplification of cDNAs from the *T. infestans* library encoding four salivary proteins.
(0.03 MB DOC)

Table S2 Antibody reactions of chicken and guinea pig sera from Bolivia to saliva and rTiSP14.6 of *T. infestans*.
(0.10 MB DOC)
