# Correction: Immunogenic Salivary Proteins of *Triatoma infestans*: Development of a Recombinant Antigen for the Detection of Low-Level Infestation of Triatomines

**DOI:** 10.1371/journal.pntd.0012484

**Published:** 2024-09-09

**Authors:** Alexandra Schwarz, Stefan Helling, Nicolas Collin, Clarissa R. Teixeira, Nora Medrano-Mercado, Jen C. C. Hume, Teresa C. Assumpção, Katrin Marcus, Christian Stephan, Helmut E. Meyer, José M. C. Ribeiro, Peter F. Billingsley, Jesus G. Valenzuela, Jeremy M. Sternberg, Günter A. Schaub

After this article [1] was published, concerns were raised about Fig 1. Specifically:

- In Fig 1A:
  ⚬ Similarities were noted between two regions in the center of the image at 47.5 and 62 kDa.
  ⚬ When brightness is adjusted, there appear to be multiple regions where no background noise could be detected which does not appear to match adjacent regions or the overall background of the panel.
- In Fig 1B, similarities were noted between multiple sets of regions at 83 and 175 kDa across the panel.
- In Fig 1C, similarities were noted between two vertical regions on the right side of the panel.

In response, author JMS stated that the above concerns are artifacts generated through reproduction of the image and that the areas of concern are not relevant to the interpretation of the respective figure. They provided underlying data from the original experiments for Fig 1 (S1–S3 Files) which do not show the similar regions listed above. The *PLOS Neglected Tropical Diseases* Editors therefore consider these concerns to be resolved.

A member of the *PLOS Neglected Tropical Diseases* Editorial Board stated any differences between S1-S5 Files and the published images would be expected for 2D-gel electrophoresis and 2D-western blots due to the methods used, and the underlying images in S1-S5 Files support the conclusions reported in [1] which do not solely rely on the gel and blot data as the findings were further validated by MS/MS.

Author JMS stated that lanes 2 and 3 in S4-S5 Files were cut from Fig 3 as they were controls not relevant to this experiment. An updated version of Fig 3 with the cut indicated by a vertical black line is provided here.

The remainder of the data underlying this article are available from author JMS.

**Fig 3:**
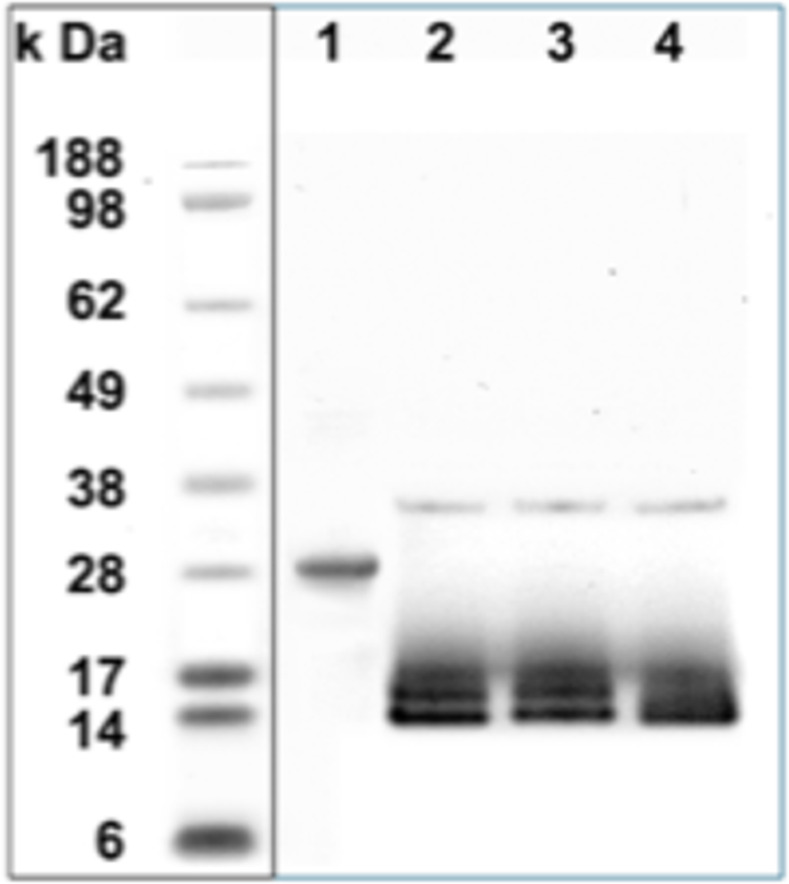
The salivary secreted protein of *T*. *infestans* (gi|149689094) after purification and deglycosylation. The His-tagged recombinant protein (r*Ti*SP14.6) was purified by HPLC (lane 1) and deglycosylated with PNGase F (lane 2), PNGase F and Sialidase A (lane 3) or PNGase F, Sialidase A and O-glycanase (lane 4). The molecular weight standards are presented on the left with the vertical line indicating the removal of two irrelevant lanes from the original gel.

## Supporting information

S1 File Underlying data supporting Fig 1A in [1]. (JPG)

S2 File Underlying data supporting Fig 1B in [1]. (JPG)

S3 File Underlying data supporting Fig 1C in [1]. (JPG)

S4 File Underlying data supporting Fig 1 in [1]. (JPG)

S5 File Underlying data supporting Fig 1 in [1]. (JPG)
