# Supplementary material for: Immunogenic Salivary Proteins of Triatoma infestans: Development of a Recombinant Antigen for the Detection of Low-Level Infestation of Triatomines
Source: PLoS Negl Trop Dis. 2009 Oct 20;3(10):e532. doi: 10.1371/journal.pntd.0000532 (PMC2760138; doi:10.1371/journal.pntd.0000532)
Supplement: Alternative Language Abstract S1 — Portuguese translation of the abstract by Dr. Eloi S. Garcia (0.02 MB DOC) [file pntd.0000532.s001.doc]

**Sumário**

**Conhecimento prévio**

Triatomíneos são vetores de *Trypanosoma cruzi*, o agente etiológico da doença de Chagas. Em grande parte da América Latina, o vetor mais efetivo, *Triatoma infestans*, tem sido controlado com sucesso por pulverização de inseticidas. Os programas de vigilância, embora raramente realizados, são necessários para investigar infestações novas e estimar a intensidade da infestação de triatomíneos no ambiente domiciliar e peridomiciliar. Considerando a possibilidade de que hospedeiros expostos a triatomíneos desenvolvam respostas imunes a antígenos da saliva, essas respostas podem ser avaliadas como marcador epidemiológico para detectar a infestação de *T. infestans*.

**Metodologia/ Dados relevantes**

As proteínas da saliva de *T. infestans* foram separadas por eletroforese 2D e sua imunogenicidade foi testada por imunoblotting utilizando soro de galinhas e cobaias expostas experimentalmente ao *T. infestans*. A partir de cinco pontos altamente imunogênicos, oito proteínas foram identificadas utilizando espectrometria de massa acoplada a nano cromatografia líquida de alta performance (liquid chromatography-Electrospray-Tandem Mass Spectrometry-nanoLC-ESI-MS/MS) e comparação das seqüências obtidas com as de uma biblioteca de cDNA de glândulas salivares de *T. infestans* (sequências expressas clonadas unidirecionalmente) e com o banco de dados NCBI. Uma proteína salivar com massa molecular de 14,6 kDa [gi|149689094] foi produzida na forma recombinante (r*Ti*SP14,6) em um sistema de expressão de células de mamíferos e reconhecida pelos soros de todos os animais testados. A especificidade da reação a r*Ti*SP14,6 for confirmada pela não reatividade a anticorpos produzidos contra a saliva de mosquito ou de flebótomos. Entretanto, essa proteína foi reconhecida pelos soros de galinhas expostas a quatro outras espécies de triatomíneos, *Triatoma brasiliensis*, *T. sordida, Rhodnius prolixus, Panstrongylus megistus* e por soros de galinhas de uma área endêmica de *T. infestans* e doença de Chagas na Bolívia.

**Conclusões/Significância**

O recombinante r*Ti*SP14,6 é um marcador epidemiológico apropriado e promissor para detecção de números pequenos de espécies diferentes de triatomíneos e poderia ser utilizado para o desenvolvimento de novas ferramentas para programas de vigilância, especialmente para corroborar a eliminação do vetor nas campanhas de controle de vetores na doença de Chagas.
