# Supplementary material for: Immunogenic Salivary Proteins of Triatoma infestans: Development of a Recombinant Antigen for the Detection of Low-Level Infestation of Triatomines
Source: PLoS Negl Trop Dis. 2009 Oct 20;3(10):e532. doi: 10.1371/journal.pntd.0000532 (PMC2760138; doi:10.1371/journal.pntd.0000532)
Supplement: Alternative Language Abstract S2 — Spanish translation of the abstract by Dr. Ricardo E. Gürtler. (0.02 MB DOC) [file pntd.0000532.s002.doc]

**Abstract**

**Antecedentes**

Los triatominos son vectores de *Trypanosoma cruzi*, el agente etiológico de la enfermedad de Chagas en América. El vector más efectivo, *Triatoma infestans*, ha sido exitosamente controlado en gran parte de América Latina por medio de la aplicación residual de insecticidas. Aunque son raramente llevados a cabo, los programas de vigilancia son necesarios para detectar nuevas infestaciones y estimar la intensidad de las infestaciones por los triatominos en habitats domésticos y peridomésticos. Debido a que los hospedadores expuestos a los triatominos desarrollan una respuesta inmune a los antígenos presentes en la saliva del vector, estas respuestas pueden ser evaluadas en cuanto a su utilidad como marcadores epidemiológicos para detectar infestaciones por *T. infestans*.

**Metodología/Principales Hallazgos**

Se separaron las proteínas salivales de *T. infestans*  mediante electroforesis en dos dimensiones y se evaluó su inmunogenicidad mediante *western blotting* usando sueros de gallinas y de cobayos que habían sido expuestos experimentalmente a *T. infestans*. De un total de cinco manchas de proteínas altamente inmunogénicas, se identificaron ocho proteínas salivales por nano Cromatografía Líquida-Ionización por Electrospray-Espectrometría de Masa en Tandem (nanoCL-IES-EM/EM) y se las comparó con *expressed sequence tags* (EST) de librerías de ADNcopia clonados unidireccionalmente a partir de glandula salival de *T. infestans* y con la base de datos NCBI. Se produjo la proteína salival de 14,6 kDa [gi|149689094] como proteína recombinante (r*Ti*SP14.6) en un sistema de expresión basado en células de mamífero reconocidas por todos los sueros animales. Se confirmó la especificidad de r*Ti*SP14.6 por la ausencia de reactividad contra anticuerpos antisaliva de mosquitos y flebotominos. Sin embargo, esta proteína fue reconocida por sueros de gallinas que habían sido expuestas a cuatro especies de triatominos, *Triatoma brasiliensis, T. sordida, Rhodnius prolixus, Panstrongylus megistus*, y por sueros de gallinas de un área endémica para *T. infestans* y la enfermedad de Chagas en Bolivia.

**Conclusiones/Significancia**

La proteína recombinante r*Ti*SP14.6 es un marcador epidemiológico apropiado y prometedor para la detección de pequeños números de triatominos de diferentes especies, y podría ser desarrollada para ser usada como una nueva herramienta en los programas de vigilancia, especialmente para corroborar la eliminación del vector en las campañas de control de la enfermedad de Chagas.
