# Supplementary material for: Immunogenic Salivary Proteins of Triatoma infestans: Development of a Recombinant Antigen for the Detection of Low-Level Infestation of Triatomines
Source: PLoS Negl Trop Dis. 2009 Oct 20;3(10):e532. doi: 10.1371/journal.pntd.0000532 (PMC2760138; doi:10.1371/journal.pntd.0000532)
Supplement: Table S1 — Primer sequences used for PCR amplification of cDNAs from the T. infestans library encoding four salivary proteins. (0.03 MB DOC) [file pntd.0000532.s003.doc]

Supplementary data

**Table S1.** Primer sequences used for PCR amplification of cDNAs from the *T. infestans* library encoding four salivary proteins.

| Salivary  protein  of  *T. infestans* | Spot  no./  protein  no. | Forward primer (5’-3’) | Reverse primer (5’-3’)* |
| --- | --- | --- | --- |
| Truncated  79 kDa  salivary apyrase  precursor  (gi|148468017) | 2/4 | TTTATAGCTGCATTACCATTTGAT | AGTTTGCTTGTCAGTTGTTA |
| Truncated  unknown  salivary protein  (gi|148468913) | 3/5 | AGCGATTTCTTCCATTTACGGTTG | GCCAAAGAAGTCCTTCAATTTTTGA |
| Salivary  lipocalin  (gi|149898816) | 4/6 | CAAACGACCGGATGCCAGCTGCCGT | ATACACAAGTTTTGGAAATCCGTTA |
| Salivary  secreted  protein  (gi|149689094) | 4/7 | TGGGCACCTCACCAATACGGTAAT | ATAAATACGTCCAAAAATGGTGA |

*Reverse primers were extended by a six histidine-tag plus a stop codon to aid purification after heterologous protein expression.
