# Supplementary material for: Immunogenic Salivary Proteins of Triatoma infestans: Development of a Recombinant Antigen for the Detection of Low-Level Infestation of Triatomines
Source: PLoS Negl Trop Dis. 2009 Oct 20;3(10):e532. doi: 10.1371/journal.pntd.0000532 (PMC2760138; doi:10.1371/journal.pntd.0000532)
Supplement: Table S2 — Antibody reactions of chicken and guinea pig sera from Bolivia to saliva and rTiSP14.6 of T. infestans. (0.10 MB DOC) [file pntd.0000532.s004.doc]

**Table S2.** Antibody reactions of chicken and guinea pig sera from Bolivia to saliva and r*Ti*SP14.6 of *T. infestans*.

| Village | Household* | *T. infestans* infestation level | *T. infestans* host | Mean O.D. of  *T. infestans* saliva as antigen^#^ | Mean O.D. of  *T. infestans* r*Ti*SP14.6 as antigen^#^ |
| --- | --- | --- | --- | --- | --- |
| Sipe Sipe | 1 | Low | Guinea pig | 0.030 | 0.000 |
|  |  |  |  | 0.179 | 0.301 |
|  |  |  |  | 0.115 | 0.089 |
|  |  |  |  | 0.073 | 0.099 |
|  |  |  |  | 0.037 | 0.000 |
|  | 2 |  |  | 0.024 | 0.026 |
|  |  |  |  | 0.020 | 0.000 |
|  |  |  |  | 0.029 | 0.090 |
|  |  |  |  | 0.029 | 0.098 |
|  |  |  |  |  |  |
|  | 1 | High | Guinea pig | 1.094 | 0.055 |
|  |  |  |  | 0.742 | 0.000 |
|  |  |  |  | 0.474 | 0.066 |
|  | 2 |  |  | 0.309 | 0.019 |
| Lipez | 1 | Low | Chicken | 0.193 | 0.043 |
|  |  |  |  |  |  |
|  | 2 | High | Chicken | 0.329 | 0.498 |
|  | 3 |  |  | 0.355 | 1.546 |
|  | 4 |  |  | 0.602 | 0.322 |
|  |  |  |  |  |  |
|  | 1 | Low | Guinea pig | 0.010 | 0.010 |
|  |  |  |  | 0.035 | 0.018 |
|  | 3 |  |  | 0.118 | 0.078 |
|  | 5 |  |  | 0.096 | 0.104 |
|  |  |  |  | 0.117 | 0.055 |
|  |  |  |  | 0.077 | 0.248 |
|  |  |  |  |  |  |
|  | 3 | High | Guinea pig | 1.727 | 0.409 |
|  |  |  |  | 1.625 | 0.121 |
|  |  |  |  | 1.553 | 0.106 |
| Chajra Corral | 1 | Low | chicken | 0.156 | 1.723 |
|  | 2 |  |  | 0.094 | 1.127 |
|  | 3 |  |  | 0.079 | 0.760 |
|  |  |  |  |  |  |
|  | 2 | High | Chicken | 0.356 | 1.391 |
|  | 3 |  |  | 0.728 | 1.717 |
|  |  |  |  | 0.523 | 0.724 |
|  |  |  |  | 0.906 | 1.132 |
| Pampas | 1 | Low | Chicken | 0.179 | 0.566 |
|  |  |  |  | 0.218 | 0.251 |
|  |  |  |  | 0.129 | 0.098 |
|  | 2 |  |  | 0.168 | 0.372 |
|  |  |  |  | 0.261 | 1.412 |
|  |  |  |  |  |  |
|  | 3 | High | Chicken | 0.228 | 1.421 |
|  |  |  |  | 0.476 | 0.819 |
| Peña Colorada | 1 | Low | Chicken | 0.129 | 0.282 |
|  |  |  |  |  |  |
|  | 1 | High | Chicken | 0.565 | 2.984 |
|  |  |  |  | 0.897 | 0.895 |
|  |  |  |  | 0.768 | 0.998 |
| Arpita | 1 | Low | Chicken | 0.159 | 0.274 |
|  |  |  |  | 0.002 | 0.159 |
|  |  |  |  | 0.191 | 0.185 |
|  |  |  |  | 0.199 | 0.136 |
|  | 2 |  |  | 0.187 | 0.665 |
|  |  |  |  |  |  |
|  | 1 | High | Chicken | 1.149 | 2.203 |
|  | 2 |  |  | 1.149 | 1.193 |
|  |  |  |  | 0.350 | 0.348 |

*Animal sera were collected in different households with a low and high infestation level of triatomines in the Department of Cochabamba from September to November in 2007.

^#^The serum reactivity (1:100 dilution) to salivary proteins was tested in ELISA assays and the mean optical density (O.D._492nm_) calculated from the results of two experiments in triplicate wells, after subtracting the O.D._492nm_ of the negative control from the final O.D..
