# Supplementary figures and images for: Correction: Immunogenic Salivary Proteins of Triatoma infestans: Development of a Recombinant Antigen for the Detection of Low-Level Infestation of Triatomines
Source: PLoS Negl Trop Dis. 2024 Sep 9;18(9):e0012484. doi: 10.1371/journal.pntd.0012484 (PMC11383227; doi:10.1371/journal.pntd.0012484)

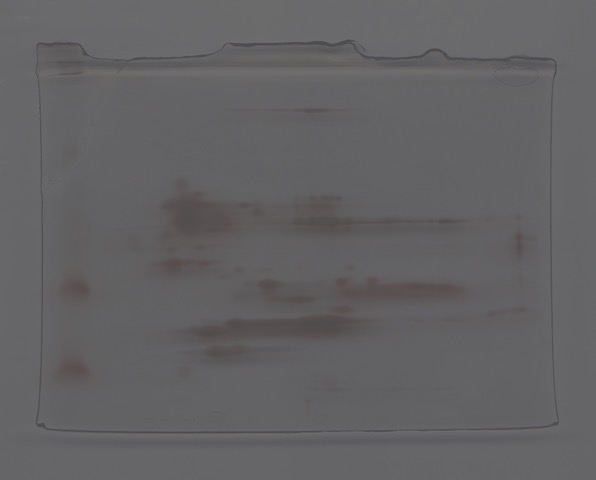

Supplement: S1 File — (JPG) [file pntd.0012484.s001.jpg]

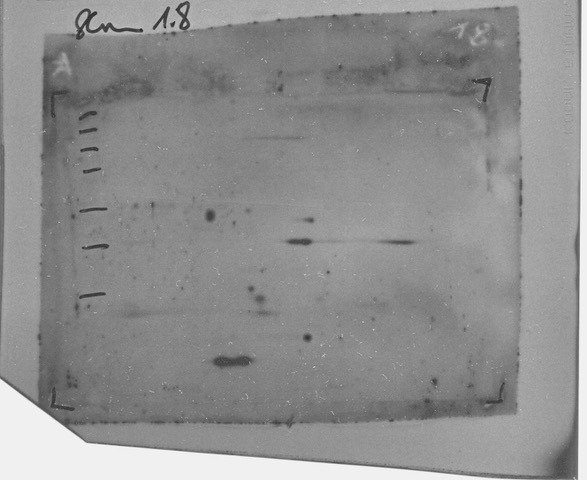

Supplement: S2 File — (JPG) [file pntd.0012484.s002.jpg]

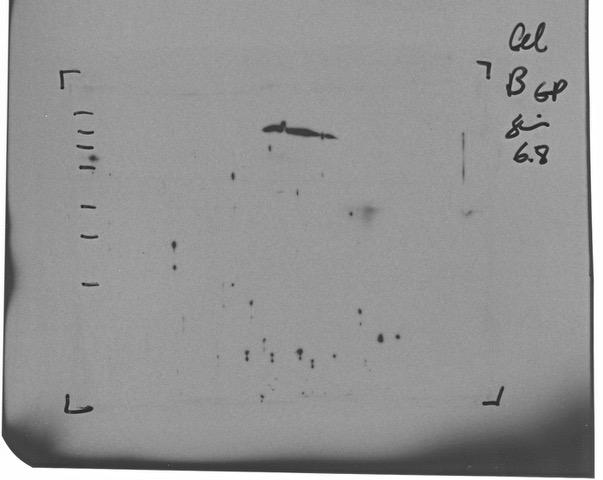

Supplement: S3 File — (JPG) [file pntd.0012484.s003.jpg]

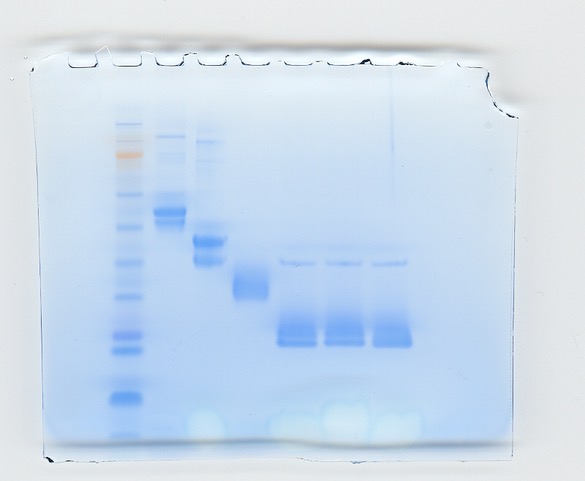

Supplement: S4 File — (JPG) [file pntd.0012484.s004.jpg]

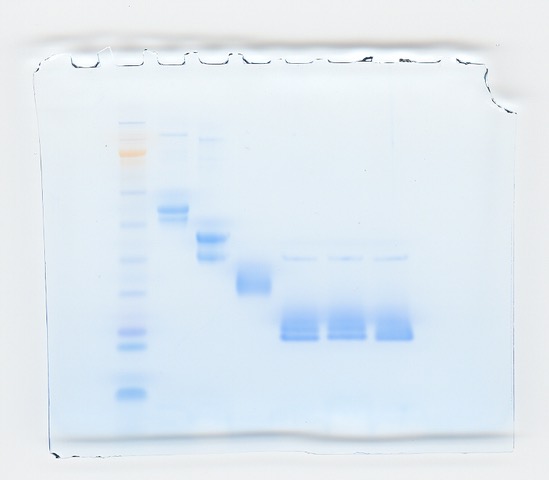

Supplement: S5 File — (JPG) [file pntd.0012484.s005.jpg]
